# An *in silico* Approach for Integrating Phenotypic and Target‐based Approaches in Drug Discovery

**DOI:** 10.1002/minf.201900096

**Published:** 2019-10-22

**Authors:** Hiroaki Iwata, Ryosuke Kojima, Yasushi Okuno

**Affiliations:** ^1^ Graduate School of Medicine Kyoto University 53 Shogoin-kawaharacho Sakyo-ku Kyoto 606-8507 Japan; ^2^ Medical Sciences Innovation Hub Program, RIKEN Cluster for Science Technology and Innovation Hub, Tsurumi-ku Kanagawa 230-0045 Japan; ^3^ Foundation for Biomedical Research and Innovation at Kobe Center for Cluster Development and Coordination, Chuo-ku, Kobe Hyogo 650-0047 Japan

**Keywords:** target deconvolution, polypharmacology, phenotypic approach, target-based approach, machine learning, drug discovery

## Abstract

Phenotypic and target‐based approaches are useful methods in drug discovery. The phenotypic approach is an experimental approach for evaluating the phenotypic response. The target‐based approach is a rational approach for screening drug candidates targeting a biomolecule that causes diseases. These approaches are widely used for drug discovery. However, two serious problems of target deconvolution and polypharmacology are encountered in these conventional experimental approaches. To overcome these two problems, we developed a new *in silico* method using a probabilistic framework. This method integrates both the phenotypic and target‐based approaches to estimate a relevant network from compound to phenotype. Our method can computationally execute target deconvolution considering polypharmacology and can provide keys for understanding the pathway and mechanism from compound to phenotype, thereby promoting drug discovery.

## Graphical abstract

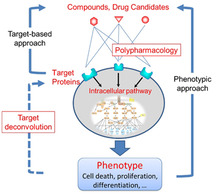

## 1 Introduction

**Figure 1:**
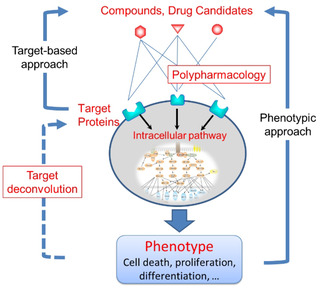
The phenotypic and target‐based approaches, target deconvolution, and polypharmacology in drug discovery

Phenotypic and target‐based approaches, developed in chemical biology, are useful methods for drug discovery^1^ (Figure 1). They are also utilized as an Adverse Outcome Pathway Framework in the field of toxicology.^2^ The phenotypic approach is an experimental approach involving techniques such as cell‐based assay and in vivo assay for evaluating the phenotypic response of cells or tissues by chemical compounds. These assays are used in drug discovery to search for active compounds that induce phenotypic responses that improve the disease state of cells or tissues.^3^ On the other hand, the target‐based approach is a rational approach for screening drug candidates targeting a biomolecule that causes diseases.1b, 4 In recent years, with the advancement of high throughput experimental technologies, the target‐based approach has become used widely for drug discovery.1a, 1b, 5

However, in drug discovery, we have two serious problems, target deconvolution and polypharmacology, that cannot be solved by the phenotypic and target‐based approaches1b, 3b, 6 (Figure 1). Target deconvolution is used to identify a target biomolecule responsible for a phenotypic response. In the phenotypic approach, even if we successfully identify an active compound that affects a target phenotype, it is not possible to reveal the target biomolecule on which the hit compound directly acts, and the relation between the target biomolecule and the phenotype.3b, 6a In addition, because the target‐based approach is a method for searching for compounds that act directly on a known target biomolecule, it cannot be applied if the target biomolecule is unknown. Thus, target deconvolution is a bottleneck in phenotypic and target‐based approaches to drug discovery. Several experimental methods have been developed in the chemical biology and chemical genetics research fields to address this problem.^7^ *In silico* approaches have been also developed such as protein‐ligand docking,^8^ virtual screening of small ligands^9^ and inverted virtual screening methods.^10^ These methods have a limitation in that they require available target protein structures.

The second critical issue in drug discovery is polypharmacology, which means that “many drugs do not act on only a single target biomolecule, but acts on multiple target biomolecules and affects phenotypes such as efficacy and toxicity”.^6^ To consider polypharmacology, it is necessary to achieve a target deconvolution for a plurality of target biomolecules that may affect drug efficacy and toxicity. However, it is extremely difficult to perform target deconvolution experiments for multiple biomolecules from the viewpoint of labor and cost. Furthermore, even if target deconvolution for multiple biomolecules can be achieved, it is very hard to design and synthesize a chemical compound that interacts with multiple target molecules by experiment.

To overcome these two problems i. e., target deconvolution and polypharmacology, we developed a new *in silico* method using the probabilistic framework. This method is based on a machine learning technique that integrates data from compound‐target protein interactions obtained from the target‐based approach and data from compound‐phenotype associations obtained from the phenotypic approach. It estimates a relevant network from compound to phenotype via target proteins. Specifically, the method consists of the following two steps. In the first step, we infer a plurality of protein candidates a compound can target by using a prediction model that is trained on the data of the compound‐target protein interactions. In the second step, we select the target proteins related to a phenotype from protein candidates predicted in the first step model using a lasso model constructed by learning the data of compound‐phenotype associations. Therefore, we can deduce a plurality of target proteins that can be interacted with by a specific compound and can be related to the phenotypic response. This is the first method for computationally executing target deconvolution considering polypharmacology, which has been unsolved by previous experimental approaches. This method could provide keys for understanding the pathway and molecular mechanism from compound to phenotype, thus promoting drug discovery

## 2 Materials and Methods

### 2.1 Dataset

We extracted 789,708 compounds that interact with 1,103 target proteins from six families, including G‐protein‐coupled receptor (GPCR), kinase, ion channel, transporter, a nuclear receptor, and protease from the ChEMBL database.^11^ We used compound‐protein pairs with a binding affinity of less than 30 μM (at Ki, EC50, and IC50) as active interaction pairs.^12^ We defined compound‐protein pairs below the set potency threshold (30uM) as non‐interaction pairs. We then prepared a data set classified for each protein family and used these data sets as gold standard data of compound‐protein interactions (CPIs) in cross‐validation (CV) experiments to evaluate the performance of the CPI prediction (Table 1).

**Table 1:** Dataset of compound‐protein interactions.

	Number of inter‐ actions	Number of non‐ interactions	Number of compounds	Number of proteins
GPCR	312,989	4,435	227,846	2033
kinase	245,853	7,578	72,736	392
ion channel	37,773	2,233	24,139	122
transporter	18,392	1,268	11,561	50
nuclear receptor	49,619	7,763	41,857	28
protease	92,958	9,001	54,778	182

We extracted phenotypes that had both more than 100 active compounds as well as inactive compounds from the PubChem database.^13^ Consequently, we obtained 34,959,972 compound‐phenotype associations (CPAs), including 900,688 compounds and 548 phenotypes. These phenotypes were classified via various assay types (e. g., In vivo, In vitro, Biochemical, Cell‐based, and Toxicity). We used the CPAs as a gold standard data in CV experiments to evaluate the performance of the CPAs prediction (Table 2 and Supplementary Table S1).

**Table 2:** Dataset of compound‐phenotype associations.

Number of associations	Number of non‐ associations	Number of compounds	Number of phenotypes
740,147	34,219,825	900,688	548

### 2.2 An *in silico* Method Using Probabilistic Framework

**Figure 2:**
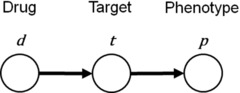
Bayesian network representation of the integrating phenotypic and target‐based approaches

To formulate the integrating phenotypic and target‐based approaches, we generated a probabilistic interpretation for the model. Figure 2 shows a Bayesian network representation for integrating the phenotypic and target‐based approaches. We consider three kinds of probabilities to model the relations between drugs, targets, and phenotypes.

First, $P\left(t\right|d)$ is a conditional probability of a binary vector, t that represents the activities of targets related to a drug with a feature vector, d . This probability is defined by a probabilistic model. Though probabilistic models can be separated into generative models and discriminative models, this type of probability can be modelled using a discriminative model such as logistic regression.

$$P\left(t_{k}=1\right|d)=\sigma \left(w_{k}d+b_{k}\right)$$

where $t=\left(t_{1},\;t_{2},\cdots ,t_{k},\;\cdots ,t_{K}\right)$ , $w_{k}$ represents a weight parameter matrix, and $b_{k}$ is a bias‐parameter vector. Since $t_{k}$ is a binary, its expectation $\bar{t_{k}}$ can be computed as follows:

$$\bar{t_{k}}\;=\sigma \left(w_{k}d+b_{k}\right).$$

Next, $P\left(p|t\right)$ is a conditional probability of a binary vector, p that represents the activities of a phenotype given a binary vector for targets,t . This probability can also be defined by a discriminative model.

Finally, $P\left(p|d\right)$ is the probability of a binary vector, p that represents phenotypes related to a drug given a feature vector, d of the drug.

Let us consider the training parameters related to $P\left(t\right|d)$ and $P\left(p|t\right)$ from the given datasets: $\left(d,t\right)\in D_{d-t}$ and $\left(d,p\right)\in D_{d-p}$ .

$P\left(t\right|d)$ can be trained using $D_{d-t}$ by the conventional manner for discriminative models like logistic regression. In contrast, to train $P\left(p|t\right)$ from $D_{d-p}$ , the following equation is considered:

$$P\left(p|d\right)=\sum_{t}P\left(p|t\right)P\left(t|d\right)=E_{t|d}\left[P\left(p|t\right)\right].$$

This probability requires the consideration of all combinations related to the targets, which requires exponential time to compute. An effective approach to such models is a mean‐field approximation that is realized by replacing the effects from variables with an expectation of them. By using this approximation, the equation above can be rewritten as

$$P\left(p|d\right)≅\;P\left(p|\bar{t}\right)$$

where $P\left(p|\bar{t}\right)$ is defined by replacing t in a model of $P\left(p|t\right)$ with its expectation $\bar{t}$ , computed by a given drug feature vector, d . Since $P\left(p|d\right)$ is given as an empirical distribution of data, $P\left(p|\bar{t}\right)$ can be trained using the KL‐divergence between the LHS and RHS probabilities in this formula. The minimization of KL‐divergence can be regarded as an optimization of the cross‐entropy error as follows:

$$KL=\sum_{p}P\left(p|d\right)\log P\left(p|\bar{t}\right)/P\left(p|d\right)=CE-H_{0}$$

where $H_{0}$ is the entropy related to $P\left(p|d\right)$ , which does not depend on parameters, and CE represents cross‐entropy error. The model, $P\left(p|\bar{t}\right)$ can be trained using the same supervised training technique by using input, $\bar{t}$ and supervised label, p .

### 2.3 Prediction of Compound‐protein Interactions and Compound‐phenotype Associations

To predict all possible compounds interacting with each protein in the six families, we proposed a method based on linear logistic regression, which is one of the machine learning methods that refers to the CGBVS method.^12^, ^14^ Compounds were represented by 894‐dimensional descriptors, called DRAGON descriptor (Version. 6.0‐2014‐Talete srl, Milano, Italy), and proteins were represented by 1,080‐dimensional descriptors, called PROFEAT descriptor.^15^ To predict compounds that are associated with a phenotype, we proposed a prediction method based on linear logistic regression. Compounds are represented by a 1,103‐dimensional binary vector, whose elements respectively use 1 or 0 to encode the interaction or un‐interaction of each protein.

To predict the possibility of CPIs or CPAs, we used a linear logistic regression with L1‐ or L2‐regression as classifier, and adopted the LIBLINEAR suite of programs^16^ (http://www.csie.ntu.edu.tw/∼cjlin/liblinear). To select the penalty parameter C, we examined various values (0.0001, 0.001, 0.01, 0.1, 1, 10, 100, 1,000, 10,000). The value that yields the highest area under the receiver operating characteristic curve score in the 5‐fold CV experiment is selected.

### 2.4 Cross‐validation of the Prediction Models

To evaluate the performance of classifiers of the CPIs or CPAs, we performed 5‐fold CV experiments using the gold standard datasets. First, we divided the gold standard dataset into five subsets. Second, we used a subset as an evaluating set and used the remaining four subsets as a learning set and constructed a prediction model using the learning set. Finally, we applied the prediction model to all pairs of the evaluating set and calculated the prediction scores.

We evaluated the performance of the prediction accuracy using a receiver operating characteristic (ROC) curve. The ROC curve is a plot of the true positive rate against the false positive rate. To evaluate the performance of the proposed methods, we calculated the area under the ROC curve (AUC), which yields a score of 1 for perfect prediction and 0.5 for random prediction.

### 2.5 Selection of Target Proteins by the Predictive Model

To select statistically significant target proteins for a phenotype, we performed feature selection from the prediction model using the following procedure (Supplementary Figure S1): (i) construction of the prediction model using all the 1,103 target proteins; (ii) calculation of the AUC score through a 5‐fold CV experiment using the gold standard dataset for the CPAs; (iii) selection of proteins that had a higher weight than the threshold (initially 0); (iv) construction of the prediction model using selected target proteins that had a higher weight than the threshold in the previous procedure; (v) calculation of the AUC scores using the prediction model constructed in the previous procedure; (vi) evaluating whether the AUC was less than 95 % of the original AUC score. We raised the threshold by 0.02 for each cycle from 0 and evaluated prediction accuracy using the selected proteins by the 5‐fold CV and calculated the AUC score. The selected proteins were defined as statistically significant proteins if the AUC score was less than 95 % of the AUC score of all proteins. The selected target proteins were important to their phenotype at that time. Significant proteins for each phenotype were selected using these analysis procedures.

### 2.6 Enrichment Analyses of the Selected Proteins

The gene ontology biological process term, enrichment analysis, was performed using statistically significant proteins.^17^ We performed a statistical overrepresentation test of the selected proteins using the PANTHER website^18^ (http://www.pantherdb.org/).

## 3 Results and Discussion

### 3.1 Overview of an *in silico* Approach Integrating Phenotypic and Target‐based Approaches

**Figure 3:**
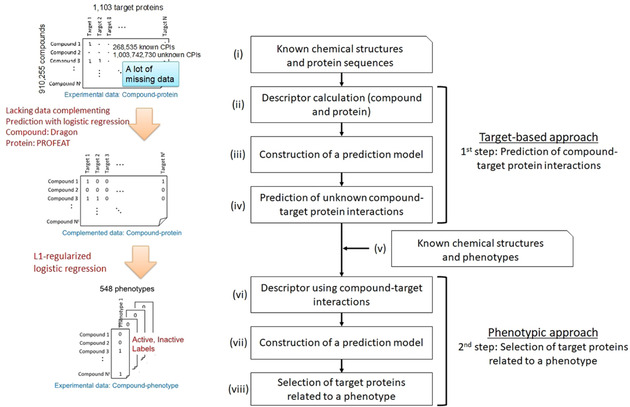
Workflow of the proposed method. Our proposed method comprises of two steps. In the first step, the method predicts compound‐target protein interactions. In the second step, the method selects target proteins related to a phenotype.

We proposed a new *in silico* method using the probabilistic framework that aims at target deconvolution considering polypharmacology in drug discovery. Figure 3 shows the workflow of the proposed method; it comprises two steps, corresponding to the target‐based and phenotypic approaches (Figure 1). In the first step (corresponding to the target‐based approach), the procedure for the prediction of CPIs is divided into the following four steps: (i) acquisition of known CPIs from the ChEMBL database^11^ (Table 1); (ii) calculation of the compound and protein feature vectors (descriptors); (iii) construction of CPIs prediction model using known interaction information to infer unknown data due to lack of CPIs; (iv) prediction of unknown CPIs using the constructed prediction model. In the second step (corresponding to the phenotypic approach), the procedure for the selection of the target proteins related to a phenotype is divided into the following four steps: (v) acquisition of known CPAs from the PubChem database^13^ (Table 2); (vi) using the CPIs as feature vectors (descriptors); (vii) construction of the prediction model to predict CPAs using the descriptors; (viii) selection of target proteins related to a phenotype.

### 3.2 Performance Evaluation of the Compound‐protein Prediction Models

Though CPI information is registered in databases, many CPIs still remain unknown. In the first step of the proposed method, we constructed a CPI prediction model to predict unknown interaction information (Figure 3). To evaluate the performance of the CPI prediction model using L1‐ and L2‐regularized logistic regression algorithms, we employed the gold standard data from the ChEMBL database for the CPIs. We performed 5‐fold CV experiments.

**Figure 4:**
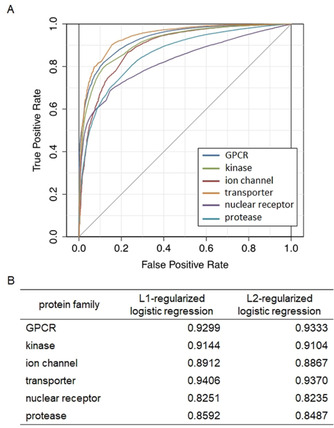
ROC curves and AUC scores from the cross‐validation experiments of compound‐protein interactions. (A) The plot shows the ROC curve; the x‐axis indicates false positive rate and the y‐axis indicates true positive rate (blue: GPCR, red: ion channel, green: kinase, purple: protease, light blue: transporter). (B) The results of AUC scores by L1‐ and L2‐regularized logistic regression are shown for each protein family.

Figure 4 shows the ROC curve (Figure 4A) and the area under the ROC curve (AUC) score (Figure 4B) in the 5‐fold CV experiments for the gold standard data for CPIs in each protein family using L1‐ and L2‐regularized logistic regression algorithms. In all protein families except the GPCR family, the AUC score for the L1‐regularized logistic regression algorithm was higher than that of the L2‐regularized logistic regression algorithm. The GPCR family and the transporter family showed high values of AUC scores (0.9299 and 0.9406, respectively). The nuclear receptor family had the lowest AUC score (0.8251) among the six families. Our proposed methods exhibited high prediction accuracy because the AUC scores for all protein families were higher than 0.8. These results suggest that the constructed prediction models had high accuracy for CPIs prediction.

### 3.3 Performance Evaluation of the Compound‐phenotype Prediction Models

In the second step of the proposed method, we constructed a CPA predictive model to predict unknown association information. We used the interactions of the target protein as a feature vector of a compound and predicted potential CPIs using the constructed prediction model. To evaluate the performance of the proposed method comparing a random method, we performed 5‐fold CV using the gold standard data for CPAs from the PubChem database.

**Figure 5:**
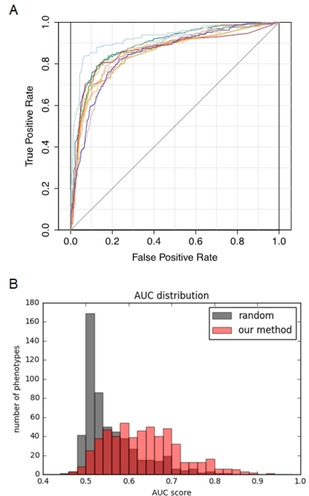
ROC curves and AUC scores for cross‐validation experiments of compound‐phenotype associations using the proposed method and random method. (A) The histogram shows the AUC scores of 548 phenotypes. The red bars represent results obtained using the proposed method while the blue bars represent results from the random method. The red bars represent results of our method while the gray bars represent results of the random method. The x‐axis indicates AUC scores while the y‐axis indicates the number of phenotypes. (B) The ROC curves for the top 10 results of the AUC scores are shown. The x‐axis indicates false positives rate while the y‐axis indicates true positives rate.

Figure 5 shows the results of the evaluation for each phenotype using the 5‐fold CV experiment. Figure 5A shows ROC curves of the top 10 AUC phenotypes while Figure 5B shows the AUC score distribution of the CPA prediction models. Details of all predictive results are shown in Supplementary Table S2. In the random method, the prediction results were shown to be random for nearly all the phenotypes (AUC score: 0.5). The AUC score distribution of the proposed method was shifted to a high value compared to the random method. This result suggests that integrating the phenotypic and target‐based approaches improves the performance.

### 3.4 Extraction of the Statistically Significant Proteins by a Predictive Model

We applied L1‐ and L2‐regularized logistic regression model to infer target proteins from the compound‐phenotype network. In the previous section, we constructed the CPA prediction models by L1‐ and L2‐regularized logistic regression models. In each method, we inferred target protein features with positive weights in the predictive model.

**Figure 6:**
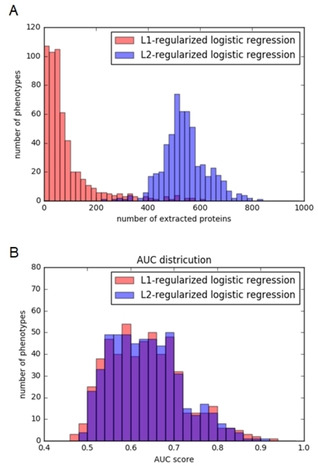
AUC scores and the number of features in the cross‐validation experiments using L1‐ and L2‐regularized logistic regression. (A) The histogram shows the number of extracted proteins for each phenotype. The x‐axis indicates the number of proteins while the y‐axis indicates the number of phenotypes. (B) The histogram shows AUC scores for each phenotype. The x‐axis shows the AUC score while the y‐axis indicates the number of phenotypes. The red bars show L1‐regularization while the blue bars show L2‐regularization.

Figure 6A shows a histogram of the number of extracted proteins (Supplementary Table S3) while Figure 6B shows a histogram of AUC scores for L1‐ and L2‐regularization (Supplementary Table S4). It was found that in most phenotypes, L1‐regularized logistic regression inferred a smaller number of features compared to L2‐regularized logistic regression (Figure 6A). This result suggests that L1‐regularization was more effective in reducing the number of proteins with positive weight. Next, histograms of the AUC scores for both the L1‐ and L2‐regularized logistic regression model exhibited a similar tendency. The average prediction accuracy of L1‐regularized regression is 0.6349 while that of L2‐regularized regression is 0.6361. These results indicate that L1‐regression predicted a smaller number of extracted proteins while maintaining analytical accuracy, compared to L2‐regression.

Furthermore, we performed additional analysis to evaluate the biological interpretation of the estimated target proteins of the phenotype. Specifically, the threshold weight was raised for each phenotype to identify more important proteins (detailed description in Methods). As a result, we achieved drastic reduction of the number of predicted target proteins, and succeeded in selecting the key proteins (from 85 proteins to 40 proteins, on average) (Supplementary Table S5). This result suggests that more important target proteins related to a phenotype were selected from the relation between the compound and the phenotype.

### 3.5 Biological Interpretation of the Inferred Target Proteins

We performed biological interpretation of the predicted target proteins through enrichment analysis using the PANTHER method.^18^ We listed gene ontology (GO) terms that were enriched in each phenotype using the GO database.^19^ Table 3 shows the list of GO biological process terms for the top 10 AUC phenotypes.

**Table 3:** List of significantly enriched GO biological process terms for the predicted protein.

Rank	AUC score	PubChem AID	PubChem description	GO term	GO description	FDR
1	0.9250	AID1828	qHTS for Inhibitors of Plasmodium falciparum proliferation: Summary	–	–	–
2	0.8910	AID1883	qHTS for differential inhibitors of proliferation of Plasmodium falciparum line W2	GO:0030154	cell differentiation	4.77E‐02
3	0.8802	AID1815	qHTS for differential inhibitors of proliferation of Plasmodium falciparum line 7G8	GO:0050896	response to stimulus	2.77E‐07
				GO:0065007	biological regulation	1.16E‐06
				GO:0007154	cell communication	3.94E‐06
				GO:0050789	regulation of biological process	1.11E‐05
				GO:0032501	multicellular organismal process	1.16E‐05
				GO:0035556	intracellular signal transduction	1.18E‐05
				GO:0044707	single‐multicellular organism process	1.20E‐05
				GO:0009987	cellular process	1.25E‐05
				GO:0007165	signal transduction	1.90E‐05
				GO:0007274	neuromuscular synaptic transmission	1.71E‐04
				GO:0003008	system process	2.03E‐04
				GO:0050790	regulation of catalytic activity	2.51E‐04
				GO:0007268	synaptic transmission	3.46E‐04
				GO:0050877	neurological system process	4.29E‐04
				GO:0007267	cell‐cell signaling	4.76E‐04
				GO:0065009	regulation of molecular function	7.00E‐04
				GO:0009719	response to endogenous stimulus	2.71E‐03
				GO:0032502	developmental process	4.10E‐03
				GO:0006796	phosphate‐containing compound metabolic process	6.52E‐03
				GO:0007270	neuron‐neuron synaptic transmission	1.04E‐02
				GO:0000165	MAPK cascade	1.05E‐02
				GO:0043066	negative regulation of apoptotic process	1.52E‐02
				GO:0006811	ion transport	1.69E‐02
				GO:0005977	glycogen metabolic process	2.88E‐02
				GO:0042592	homeostatic process	3.94E‐02
4	0.8706	AID540	Cell Viability – N2a	–	–	–
5	0.8657	AID1816	qHTS for differential inhibitors of proliferation of Plasmodium falciparum line GB4	GO:0032501	multicellular organismal process	9.95E‐04
				GO:0050789	regulation of biological process	1.34E‐03
				GO:0065007	biological regulation	1.43E‐03
				GO:0030154	cell differentiation	1.71E‐03
				GO:0019233	sensory perception of pain	1.74E‐03
				GO:0043066	negative regulation of apoptotic process	2.06E‐03
				GO:0044707	single‐multicellular organism process	2.74E‐03
				GO:0035556	intracellular signal transduction	2.77E‐03
				GO:0050790	regulation of catalytic activity	3.64E‐03
				GO:0065009	regulation of molecular function	7.14E‐03
				GO:0050896	response to stimulus	7.61E‐03
				GO:0050909	sensory perception of taste	1.02E‐02
				GO:0007165	signal transduction	1.72E‐02
				GO:0007154	cell communication	3.37E‐02
				GO:0006812	cation transport	3.39E‐02
				GO:0000165	MAPK cascade	3.57E‐02
6	0.8605	AID543	Cell Viability – H‐4‐II‐E	–	–	–
7	0.8560	AID988	Cell Viability – LYMP2‐024	–	–	–
8	0.8460	AID426	Cell Viability – Jurkat	–	–	–
9	0.8459	AID1882	qHTS for differential inhibitors of proliferation of Plasmodium falciparum line Dd2	GO:0065007	biological regulation	6.16E‐09
				GO:0050789	regulation of biological process	8.79E‐09
				GO:0032501	multicellular organismal process	1.09E‐05
				GO:0044707	single‐multicellular organism process	1.24E‐05
				GO:0007154	cell communication	1.37E‐05
				GO:0009987	cellular process	2.96E‐05
				GO:0003008	system process	1.16E‐04
				GO:0050896	response to stimulus	6.51E‐04
				GO:0035556	intracellular signal transduction	8.86E‐04
				GO:0007165	signal transduction	1.21E‐03
				GO:0050877	neurological system process	1.25E‐03
				GO:0019229	regulation of vasoconstriction	2.15E‐03
				GO:0009719	response to endogenous stimulus	5.18E‐03
				GO:0007274	neuromuscular synaptic transmission	5.45E‐03
				GO:0032502	developmental process	1.04E‐02
				GO:0006796	phosphate‐containing compound metabolic process	1.45E‐02
				GO:0001525	angiogenesis	1.46E‐02
				GO:0008015	blood circulation	1.52E‐02
				GO:0000165	MAPK cascade	1.61E‐02
				GO:0050790	regulation of catalytic activity	1.94E‐02
				GO:0043066	negative regulation of apoptotic process	2.02E‐02
				GO:0007268	synaptic transmission	2.31E‐02
				GO:0007267	cell‐cell signaling	2.33E‐02
				GO:0006874	cellular calcium ion homeostasis	2.70E‐02
				GO:0005977	glycogen metabolic process	3.26E‐02
				GO:0065009	regulation of molecular function	3.62E‐02
				GO:0007169	transmembrane receptor protein tyrosine kinase signaling pathway	4.90E‐02
				GO:0042592	homeostatic process	4.93E‐02
10	0.8456	AID1877	qHTS for differential inhibitors of proliferation of Plasmodium falciparum line D10	GO:0050789	regulation of biological process	5.79E‐07
				GO:0032501	multicellular organismal process	7.06E‐07
				GO:0044707	single‐multicellular organism process	8.95E‐07
				GO:0065007	biological regulation	1.58E‐06
				GO:0003008	system process	1.87E‐04
				GO:0050877	neurological system process	4.55E‐04
				GO:0043066	negative regulation of apoptotic process	1.82E‐03
				GO:0007154	cell communication	3.95E‐03
				GO:0035556	intracellular signal transduction	6.14E‐03
				GO:0019233	sensory perception of pain	6.16E‐03
				GO:0050896	response to stimulus	1.04E‐02
				GO:0030154	cell differentiation	1.31E‐02
				GO:0050790	regulation of catalytic activity	1.41E‐02
				GO:0007268	synaptic transmission	1.61E‐02
				GO:0007165	signal transduction	2.28E‐02
				GO:0065009	regulation of molecular function	2.65E‐02
				GO:0032502	developmental process	3.38E‐02
				GO:0050909	sensory perception of taste	4.46E‐02

Though AID1828 had the highest AUC score, no GO terms were obtained when it was tested by enrichment analysis (Table 3). For AID1888, which had the second highest AUC score, GO: 0030154 *cell differentiation* was selected by the enrichment analysis. This showed that our predicted target proteins in AID1888 could be related to cell differentiation. Since AID1888 is an inhibitors assay for the proliferation of *P. falciparum*, the result was reasonable because cell proliferation and cell differentiation are closely related. For AID1815 with the third highest AUC score, GO: 050896 *response to stimulus* was selected as the highest ranked GO term by enrichment analysis. The other previous omics analysis reported that proteins obtained from malaria life cycle stages were enriched in GO: 050896 *response to stimulus*,^20^ which is consistent with our result. In addition, the previous study also reported that the proteins obtained from the malaria life cycle were enriched in GO: 0009987 *cellular process*. In our results, GO: 0009987 *cellular process* was selected for AID1815 and AID1882 with the ninth highest AUC score. These biological interpretations suggest that our prediction model offers reasonable results.

## 4 Conclusions

We proposed an *in silico* method using the probabilistic framework that integrates both data from the phenotypic and target‐based approaches. The proposed method enables us to computationally execute target deconvolution considering polypharmacology, which cannot be solved by conventional experimental approaches. The method consists of two machine learning models. The first model predicts proteins targeted by a compound using the CGBVS model trained on the compound‐target protein interaction data. The second model selects statistically significant proteins related to a phenotype from candidate proteins predicted by the CGBVS model. To evaluate the prediction performance of the method, we applied data from the ChEMBL and PubChem databases to the models. The first model indicated a prediction performance higher than 0.8 AUC score on average for six protein families.

Using the proposed method, we inferred target proteins for assays of differential inhibitors of proliferation of *P. falciparum*. The inferred proteins were related to the cellular differentiation process and the life cycle stages of *P. falciparum*. Our approach is expected to be useful for target deconvolution considering polypharmacology in drug discovery.

## Abbreviations

CPI: compound‐protein interaction
CPA: compound‐phenotype association
CV experiment: cross‐validation experiment
ROC curve: the receiver operating characteristic
AUC: the area under the ROC curve
GPCR: G‐protein‐coupled receptor
GO: gene ontology

## Conflict of Interest

None declared.

## Supporting information

As a service to our authors and readers, this journal provides supporting information supplied by the authors. Such materials are peer reviewed and may be re‐organized for online delivery, but are not copy‐edited or typeset. Technical support issues arising from supporting information (other than missing files) should be addressed to the authors.

Supplementary

Supplementary

## References

[1a] R. Heilker , U. Lessel , D. Bischoff , Drug Discovery Today 2018; 10.1016/j.drudis.2018.10.009 30359770

[1b] G. E. Croston , Expert Opin. Drug Discovery 2017, 12, 427–429; 10.1080/17460441.2017.1308351 28306350

[1c] W. Zheng , N. Thorne , J. C. McKew , Drug Discovery Today 2013, 18, 1067–1073; 23850704 10.1016/j.drudis.2013.07.001 PMC4531371

[1d] D. Swinney , Clin. Pharmacol. Ther. 2013, 93, 299–301. 23511784 10.1038/clpt.2012.236

[2a] T. E. Allen , J. M. Goodman , S. Gutsell , P. J. Russell , Chem. Res. Toxicol. 2014, 27, 2100–2112; 25354311 10.1021/tx500345j

[2b] G. T. Ankley , R. S. Bennett , R. J. Erickson , D. J. Hoff , M. W. Hornung , R. D. Johnson , D. R. Mount , J. W. Nichols , C. L. Russom , P. K. Schmieder , J. A. Serrrano , J. E. Tietge , D. L. Villeneuve , Environ. Toxicol. Chem. 2010, 29, 730–741. 20821501 10.1002/etc.34

[3a] Y. Feng , T. J. Mitchison , A. Bender , D. W. Young , J. A. Tallarico , Nat. Rev. Drug Discovery 2009, 8, 567–578; 19568283 10.1038/nrd2876

[3b] J. G. Moffat , F. Vincent , J. A. Lee , J. Eder , M. Prunotto , Nat. Rev. Drug Discovery 2017, 16, 531–543. 28685762 10.1038/nrd.2017.111

[4] F. Samsdodd , Drug Discovery Today 2005, 10, 139–147. 15718163 10.1016/S1359-6446(04)03316-1

[5] D. C. Swinney , J. Anthony , Nat. Rev. Drug Discovery 2011, 10, 507. 21701501 10.1038/nrd3480

[6a] J. U. Peters , J. Med. Chem. 2013, 56, 8955–8971; 23919353 10.1021/jm400856t

[6b] A. L. Hopkins , J. S. Mason , J. P. Overington , Curr. Opin. Struct. Biol. 2006, 16, 127–136. 16442279 10.1016/j.sbi.2006.01.013

[7a] G. C. Terstappen , C. Schlupen , R. Raggiaschi , G. Gaviraghi , Nat. Rev. Drug Discovery 2007, 6, 891–903; 17917669 10.1038/nrd2410

[7b] L. H. Jones , M. E. Bunnage , Nat. Rev. Drug Discovery 2017, 16, 285–296; 28104905 10.1038/nrd.2016.244

[7c] H. Lee , J. W. Lee , Arch. Pharmacal Res. 2016, 39, 1193–1201; 10.1007/s12272-016-0791-z 27387321

[7d] F. Cong , A. K. Cheung , S. M. Huang , Annu. Rev. Pharmacol. Toxicol. 2012, 52, 57–78. 21819237 10.1146/annurev-pharmtox-010611-134639

[8a] L. G. Ferreira , R. N. Dos Santos , G. Oliva , A. D. Andricopulo , Molecules 2015, 20, 13384–13421; 26205061 10.3390/molecules200713384 PMC6332083

[8b] N. S. Pagadala , K. Syed , J. Tuszynski , Biophys. Rev. Lett. 2017, 9, 91–102. 10.1007/s12551-016-0247-1 PMC5425816 28510083

[9a] A. Kumar , K. Y. Zhang , Methods 2015, 71, 26–37; 25072167 10.1016/j.ymeth.2014.07.007 PMC7129923

[9b] V. Kumar , S. Krishna , M. I. Siddiqi , Methods 2015, 71, 64–70. 25171960 10.1016/j.ymeth.2014.08.010

[10a] X. Xu , M. Huang , X. Zou , Biophys. Rev. Lett. 2018, 4, 1–16; 10.1007/s41048-017-0045-8 PMC5860130 29577065

[10b] Y. Chen , D. Zhi , Proteins Struct. Funct. Bioinf. 2001, 43, 217–226. 10.1002/1097-0134(20010501)43:2<217::aid-prot1032>3.0.co;2-g 11276090

[11] A. Gaulton , L. J. Bellis , A. P. Bento , J. Chambers , M. Davies , A. Hersey , Y. Light , S. McGlinchey , D. Michalovich , B. Al-Lazikani , J. P. Overington , Nucleic Acids Res. 2012, 40, D1100–1107. 10.1093/nar/gkr777 PMC3245175 21948594

[12] M. Hamanaka , K. Taneishi , H. Iwata , J. Ye , J. Pei , J. Hou , Y. Okuno , Mol. Inf. 2017, 36. 10.1002/minf.201600045 27515489

[13] S. Kim , P. A. Thiessen , E. E. Bolton , J. Chen , G. Fu , A. Gindulyte , L. Han , J. He , S. He , B. A. Shoemaker , J. Wang , B. Yu , J. Zhang , S. H. Bryant , Nucleic Acids Res. 2016, 44, D1202–1213. 10.1093/nar/gkv951 PMC4702940 26400175

[14] H. Yabuuchi , S. Niijima , H. Takematsu , T. Ida , T. Hirokawa , T. Hara , T. Ogawa , Y. Minowa , G. Tsujimoto , Y. Okuno , Mol. Syst. Biol. 2011, 7, 472. 21364574 10.1038/msb.2011.5 PMC3094066

[15] Z.-R. Li , H. H. Lin , L. Han , L. Jiang , X. Chen , Y. Z. Chen , Nucleic Acids Res. 2006, 34, W32–W37. 16845018 10.1093/nar/gkl305 PMC1538821

[16] R.-E. Fan , K.-W. Chang , C.-J. Hsieh , X.-R. Wang , C.-J. Lin , J. Mach. Learn. Res. 2008, 9, 1871–1874.

[17] C. Gene Ontology , Nucleic Acids Res. 2015, 43, D1049–1056.

[18] H. Mi , A. Muruganujan , J. T. Casagrande , P. D. Thomas , Nat. Protoc. 2013, 8, 1551–1566. 23868073 10.1038/nprot.2013.092 PMC6519453

[19] M. Ashburner , C. A. Ball , J. A. Blake , D. Botstein , H. Butler , J. M. Cherry , A. P. Davis , K. Dolinski , S. S. Dwight , J. T. Eppig , Nat. Genet. 2000, 25, 25–29. 10802651 10.1038/75556 PMC3037419

[20] O. A. Tomescu , D. Mattanovich , G. G. Thallinger , BMC Syst. Biol. 2014, 8 Suppl 2, S4. 25033389 10.1186/1752-0509-8-S2-S4 PMC4101701

