# Supplementary material for: An in silico Approach for Integrating Phenotypic and Target‐based Approaches in Drug Discovery
Source: Mol Inform. 2019 Oct 22;39(1-2):1900096. doi: 10.1002/minf.201900096 (PMC7050533; doi:10.1002/minf.201900096)
Supplement: Supplementary file 1 — Supplementary [file MINF-39-1900096-s001.pdf]

molecular informatics

Supporting Information

© Copyright Wiley-VCH Verlag GmbH & Co. KGaA, 69451 Weinheim, 2020

# An *in silico* Approach for Integrating Phenotypic and Target-based Approaches in Drug Discovery

Hiroaki Iwata, Ryosuke Kojima, and Yasushi Okuno*© 2019 The Authors. Published by Wiley-VCH Verlag GmbH & Co. KGaA.
This is an open access article under the terms of the Creative Commons Attribution License, which permits use, distribution and reproduction in any medium, provided the original work is properly cited.

# Supporting Information for

# An in silico approach for integrating phenotypic and target-based approaches in drug discovery

Research Article

**Hiroaki Iwata[a,b], Ryosuke Kojima[a], and Yasushi Okuno*[a,b,c]**

[a] Graduate School of Medicine, Kyoto University Shogoin-kawaharacho, Sakyo-ku Kyoto 606-8507, Japan
[b] Medical Sciences Innovation Hub Program, RIKEN Cluster for Science, Technology and Innovation Hub, Tsurumi-ku, Kanagawa 230-0045, Japan
[c] Foundation for Biomedical Research and Innovation at Kobe, Center for Cluster Development and Coordination, Chuo-ku, Kobe, Hyogo 650-0047, Japan

*Corresponding Authors

Graduate School of Medicine, Kyoto University Shogoin-kawaharacho, Sakyo-ku Kyoto 606-8507, Japan
Tel: +81- 75-751-4881
Fax: +81- 75-751-4881
E-mail: okuno.yasushi.4c@kyoto-u.ac.jp

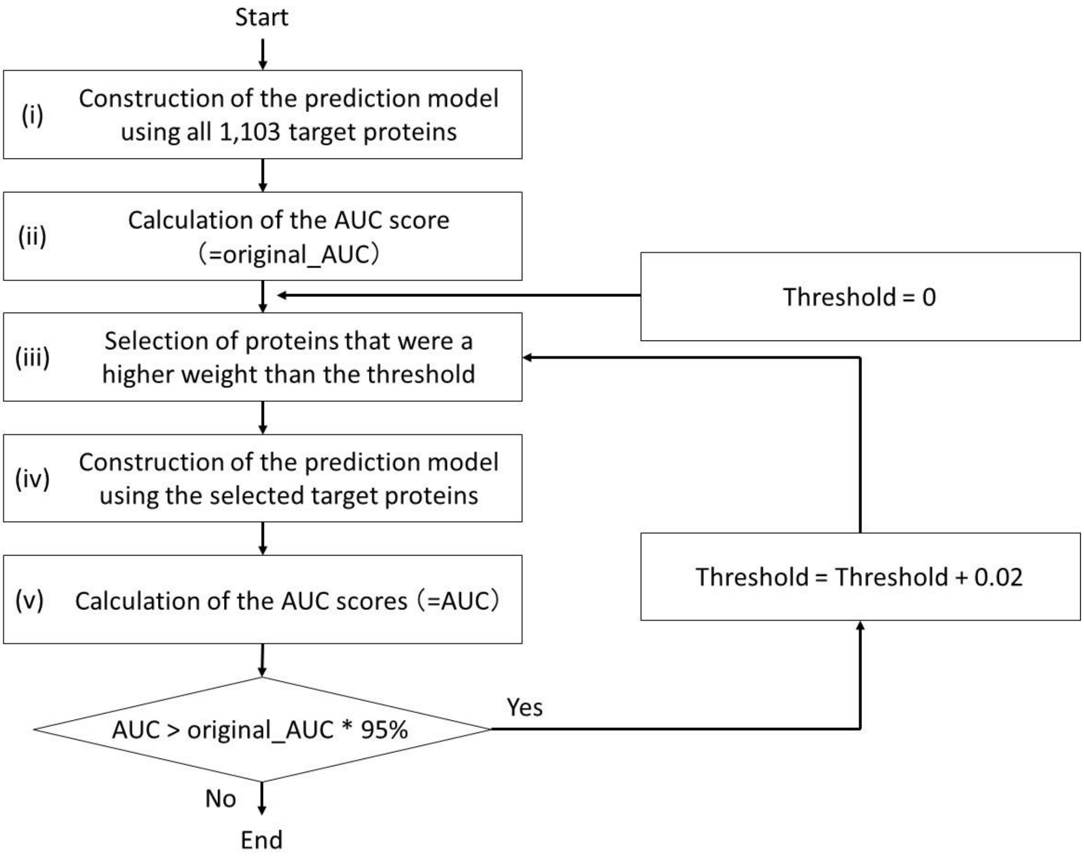


Supplementary Figure S1: Procedure for selecting statistically significant target proteins
